# Mechanical Analysis of the Quasi-Static and Dynamic Composite Action in PV Modules with Viscoelastic Encapsulant

**DOI:** 10.3390/ma17061317

**Published:** 2024-03-13

**Authors:** Chiara Bedon, Filipe A. Santos, Marco Fasan

**Affiliations:** 1 Department of Engineering and Architecture, University of Trieste, 34127 Trieste, Italy; mfasan@units.it; 2 CERIS-NOVA, Department of Civil Engineering, NOVA School of Science and Technology, Universidade NOVA de Lisboa, 2829-516 Caparica, Portugal; fpas@fct.unl.pt

**Keywords:** photovoltaic (PV) module, building integrated (BIPV) solutions, load-bearing capacity, shear coupling, sandwich section

## Abstract

The mechanical analysis of photovoltaics and building integrated photovoltaics is a key step for their optimal design and certification, and requires careful consideration, alongside solar power, durability and functionality issues. The solar cells are encapsulated in thin interlayers that are usually composed of a viscoelastic Ethylene–Vinyl Acetate compound, and protected by thin glass and/or plastic layers. This paper investigates the out-of-plane bending response of a full-scale commercial PV module and focuses attention on the shear bonding efficiency of the thin encapsulant for quasi-static and dynamic mechanical considerations. The parametric analytical analysis, carried out in this study for a laminated glass plate, highlights the possible consequences of the viscoelastic shear coupling on the cross-section load-bearing demand in the covers. As a direct effect of severe operational conditions (i.e., ageing, non-uniform/cyclic thermal gradients, humidity, extreme mechanical/thermal loads, etc.) the shear rigidity and adhesion of these films can suffer from repeated/progressive modification and even degradation, and thus induce major stress and deflection effects in the out-of-plane mechanical response of the PV module components. The minimum shear bond efficiency required to prevent mechanical issues is calculated for various configurations of technical interest. Accordingly, it is shown how the quasi-static and dynamic mechanical performance of the system modifies as a function of a more rigid or weak shear coupling.

## 1. Introduction

The mechanical performance assessment of photovoltaic (PV) modules and building integrated photovoltaics (BIPV) is a design task of major importance for the long-term functionality and optimization of commercial products [1,2]. Many aspects should be taken into account to ensure their structural efficiency [3,4,5]. To this aim, product standards impose several rigid procedural steps to verify the load-bearing capacity. Critical aspects to address for the reliability of a given PV module reliability are related to its strength against ordinary external actions, which usually takes the form of human handling, snow, wind or even hailstorms [6,7,8].

**Figure 1:**
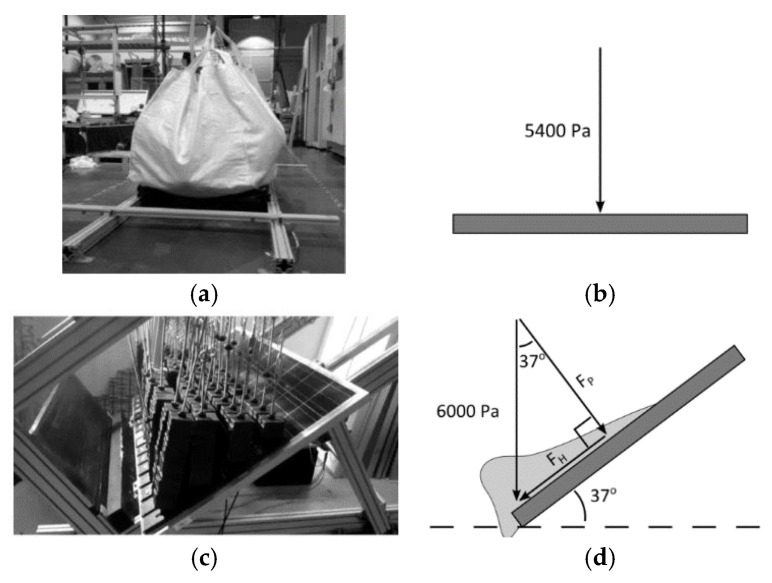
Reference arrangement for (**a**) Mechanical Loading (ML) and (**c**) Inhomogeneous Mechanical Loading (IML) test protocols on PV modules, according to IEC 61215:2021, with (**b**,**d**) schematic cross-sections of loading setup.

In order to characterize and verify the PV response against such external mechanical loads (see, for example, the IEC 61215:2021 document [9]), quality tests are required in the form of Mechanical Loading (ML), Inhomogeneous Mechanical Loading (IML) and Dynamic Mechanical Loading (DML) protocols (Figure 1). It is important to verify that the PV module components (even subjected to major stress peaks) are not affected by visual damage and severe loss of electrical functionality. However, major criticalities are expected in the solar cells [10,11]. To ensure an efficient mechanical performance, the supporting metal frame is optimally detailed to offer an important bracing contribution to the sandwich section. The local effect of fixing brackets is also important for the out-of-plane bending performance assessment of the PV module. However, basic calculations (especially for the mid-span stress and deflection analysis) can be carried out under ideal boundary conditions with a rigid frame assumption along the edges of the laminated section [4].

For the interpretation of failure mechanisms, experimental protocols can be efficiently supported and extended by refined Finite Element (FE) numerical models [12,13,14], in which the composition and layout of PV components is realistically taken into account, or even integrated by robust analytical formulations [15]. In doing so, it is important to note that thermal boundaries have certain major effects in terms of solar cell faults (i.e., hot-spotting, etc. [16]), but also implicit effects on the mechanical response of the sandwich section [17,18,19,20,21]. In this regard, an increasing number of studies have addressed the long-term power loss and functionality degradation of PV modules [22,23].

Among others, the pure mechanical performance and capacity assessment of PV modules as load-bearing components in buildings is of primary importance (see Section 2.1), not only because they are implicitly associated with the electrical functionality of the system, but also in terms of possible risk for people, in case of breakage. For this reason, for example, the PV module performance under inhomogeneous snow loads in cold temperatures (down to −30 °C) was investigated in [24], taking into account the encapsulant stiffening with extreme temperatures and the consequent stress modification in the glass cover.

Structurally speaking, typical manifestations of a possible mechanical capacity loss due to long-term effects are possibly associated with the progressive material deterioration and/or delamination of the constituent layers, which further involves a reduction of load-bearing capacity [25] with similar criticalities to laminated glass members [26,27]. This is especially the case for long-term mechanical loads or unfavourable temperatures [25]. However, there are many other influencing parameters to account for, given that the backsheet composition is also responsible for different internal temperature distributions, and thus material response [28]. Moreover, the encapsulant itself is highly sensitive to the operational vibrational frequency of the system [29,30]. Based on a consolidated simplified methodology, the present study focuses attention on the mechanical performance assessment of the PV sandwich section, and on the contribution and interaction of the thin glass and backsheet layers when subjected to quasi-static or dynamic loads, and the viscoelastic encapsulant offering a different bond. The role and capacity of the glass cover are investigated based on the effective thickness formulation in use for structural laminated glass plates [31,32]. As shown, the analysis of shear bond efficiency assumes a critical role in the bending performance assessment of the PV system. However, an appropriate analysis of mechanical performance indicators can suggest possible optimization strategies for more efficient structural performances.

## 2. Research Methods

### 2.1. Mechanical Analysis of the Sandwich Section

The present study is primarily focused on the structural analysis of single-glass PV modules typically used in buildings (Section 2.2). Most commercial PV modules are characterized by a relatively thin cross-section and a typically high slenderness, which makes them highly flexible and vulnerable to out-of-plane bending mechanical loads.

**Figure 2:**
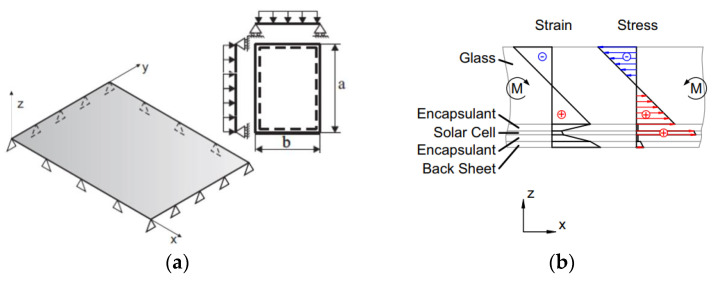
Simplified mechanical analysis of PV modules: (**a**) “SSSS” setup with uniformly distributed load; (**b**) stress distribution in the cross-section (reproduced from [1] with permission from Elsevier^©^, copyright license number 5654100960128, October 2023).

Under the assumption of simplified boundary conditions for a given PV modular unit like in Figure 2a—i.e., simple supports along the four edges (“SSSS”) and a uniformly distributed load *q*—the effect of external mechanical loads typically manifests in a classical bi-triangular internal distribution of bending stresses in glass and in the backsheet layer (Figure 2b). Such a mechanical behaviour (and its effects on the energy functionality and efficiency of PV modules) has been largely explored in the literature [1,2,3,4,5]. In addition, most of these existing mechanical studies assumed a constant shear rigidity for the encapsulant characterization, even for complex numerical simulations [8,10,11].

**Figure 3:**
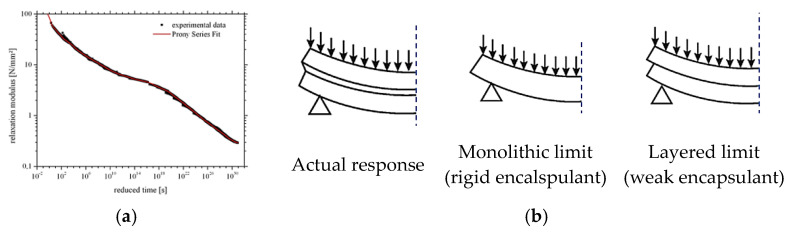
Viscoelastic encapsulant. (**a**) Typical relaxation modulus for EVA interlayer with time (reproduced from [25] under the terms and conditions of a CC-BY license agreement) and (**b**) schematic out-of-plane bending response.

Despite the use of additives, the encapsulant has a typical viscoelastic behaviour and is characterized by possible variations in shear stiffness [25,33,34,35,36,37,38], as a consequence of strain rate effects, temperature gradients, long-term phenomena, etc. (Figure 3a). From a mechanical point of view, this means that the out-of-plane response of the PV sandwich section strictly depends on the partially rigid shear bonding efficiency (Figure 3b), which varies according to operational conditions and is ideally comprised between the upper “monolithic” and lower “layered” bounds, corresponding to rigid or weak encapsulant, respectively.

### 2.2. Reference PV Module

**Figure 4:**
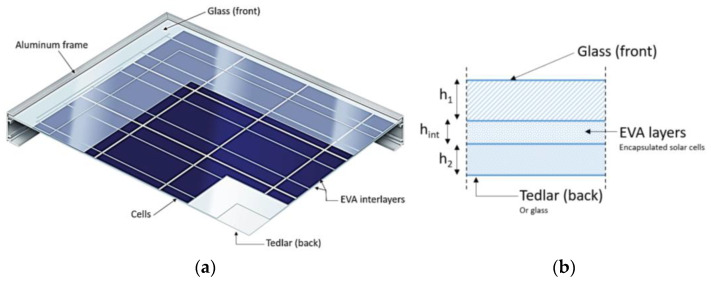
Reference PV module. (**a**) Axonometry and (**b**) schematic cross-section.

The typical commercial single-glass PV module has a sandwich section like in Figure 4 and consists of a thin glass cover on top (with thickness range *h*_1_ ≈ 3÷5 mm), a plastic backsheet (*h*_2_ ≈ 1 ÷ 2 mm) and interposed EVA films (*h_int_* ≈ 1 mm in total). The soft interlayer provides any mechanical interaction of stiffer covers (Table 1 [39,40,41]).

The present parametric analysis was carried out by taking inspiration from Figure 4, with *B* = 1.135 m × *L* = 1.75 m as the global dimensions and *h*_1_ = 3.2 mm as the thickness of glass, *h_int_* = 1 mm for EVA and *h*_2_ = 1 mm for the Tedlar^®^ backsheet. According to [4,8], for preliminary calculations, the metal frame can be considered sufficiently rigid to describe it in the form of linear supports along the four edges. Also, the electrical components (i.e., solar cells, busbars, etc.) can be disregarded for the mechanical analysis of the system [4,8].

### 2.3. Structural Analysis of the PV Composite Section

The out-of-plane bending response and mechanical capacity verification of the glass cover can be carried out according to [31,32]. Similarly to a laminated glass member, the performance optimization of the PV system under external, quasi-static mechanical loads requires that the glass cover does not suffer from high tensile stress peaks and deflections. Accordingly, the maximum effect of a design uniform pressure *q* can be expressed by means of the approximate geometric nonlinear analytical equations in use for glass, that is [31]:

(1) $$\sigma_{max}=k_{1}\frac{A}{{\hat{h}}_{1;\sigma }^{2}}q$$

and

(2) $$w_{max}=k_{4}\frac{A^{2}}{{\hat{h}}_{w}^{3}}\frac{q}{E}$$

with *A* = *B × L* being the panel size, *E* being the modulus of elasticity of glass (Table 1), *k*_1_ and *k*_4_ being the two coefficients for stress and deflection analysis [31], and ${\hat{h}}_{1;\sigma }$, ${\hat{h}}_{w}$ being the corresponding effective thicknesses (Section 2.5). The combined effect of multiple *i* = 1,…*N* actions can be generally taken into account by verifying that:

(3) $$\sum_{i=1}^{N}\frac{\sigma_{max}^{i}}{f_{g}^{i}}\leq 1$$

(4) $$w=\sum_{i=1}^{N}w_{max}^{i}\leq w_{lim}$$

For the majority of typical structural glass applications, the stress peak verification is in general the most severe verification step for such a tensile brittle material. In addition, the deflection verification can be critical for particularly slender and flexible glass components, as it is in PV modules. Also, specific performance indicators may be required.

### 2.4. Structural Performance Indicators for the Glass Cover

Based on [31] and Equations (3) and (4), the tensile stress peak at the Ultimate Limit State (from Equation (1)) should not exceed the design tensile strength of glass *f_g_*, while the deflection at the Serviceability Limit State (from Equation (2)) should be limited to *w_lim_* = *b*/60 ≈ 18 mm (with *b* the shortest edge, or in any case 30 mm).

The design tensile strength *f_g_* in Equation (3) implicitly accounts for several glass production features and quasi-static fatigue phenomena [31,42]. Depending on cross-sectional features, size, boundaries and other parameters, the design value *f_g_* should be calculated case by case [31].

The deflection limit *w_lim_* in Equation (4), which in structural glass members is defined for preservation of comfort and minimization of possible damage in finishing/secondary components, strictly depends on the mechanical boundary conditions [31].

For PV modules, a reliable *w_lim_* value should be specified to preserve the integrity of load-bearing components, but also to implicitly save the functionality of electrical components. In this regard, literature experiments on double-glass PV modules (see for example [4] and [8,43]) recorded maximum deflections up to *w_max_* ≈ 18 mm = *b*/55 (for PV samples with *h*_1_ = *h*_2_ = 3.2 mm thick glass) and *w_max_* ≈ 42 mm = *b*/23 (*h*_1_ = *h*_2_ = 2 mm), without visual detection of mechanical damage. However, the residual electrical functionality was not verified. The maximum deflection measured in [5] for double-glass PV modules was quantified as *w_max_* ≈ 25 mm = *b*/48, but the primary attention in the analysis of results was given to mechanical bending only.

A single-glass PV module (inclusive of the metal frame) was numerically explored in [44], and its mechanical response in out-of-plane bending achieved a maximum deflection of *w_max_* ≈ 25.9 mm = *b*/41. Single-glass PV samples tested in [45] achieved maximum deflections of *w_max_* ≈ 40 mm, but revealing—in most cases—minor cracks of solar cells, starting from deflections larger than ≈ 20 mm. The propagation and orientation of cracks in multi-crystalline and mono-crystalline solar cells belonging to single-glass PV modules under four-point bending mechanical loads was addressed in [1].

The analysis of experimental results emphasized a major damage propagation in the solar cells, for out-of-plane bending deformations in the order of *w_max_* ≈ 40 mm = *L*/21 (with *L* the span in the beam-like experimental setup), but revealing crack initiation for relatively lower deflections (approximately *w_max_* ≈ 18–20 mm = *L*/46), and a sensitivity of crack propagation on the orientation of solar cells.

In all recalled studies, minor attention was given to the viscoelastic behaviour of the encapsulant (i.e., by assuming a constant, equivalent shear rigidity for numerical simulations, or an equivalent fixed time step for the mechanical load application), and thus to the possible modification of the bending response (solar cells included) of the examined PV systems. Moreover, considering the high variability of geometrical and mechanical details for commercial PV modules, *w_lim_* was assumed in this study to be equal to *b*/60 [31] for comparative purposes only.

### 2.5. Analytical Modelling and Shear Bond Efficiency

According to [31,32], the mechanical response of the PV module—under the simplified assumption of linear simple supports along the four edges (Figure 2a)—can be estimated based on Equations (1) and (2), where a major advantage derives from the use of a monolithic thickness with equivalent bending characteristics for the analysis of deflections and stress peaks, respectively. The out-of-plane bending deformation of the PV sandwich plate can be calculated based on the deflection-effective thickness:

(5) $${\hat{h}}_{w}=\sqrt[{3}]{\frac{1}{\frac{\eta }{h_{1}^{3}+h_{2}^{3}+12\frac{h_{1}h_{2}}{h_{1}+h_{2}}H^{2}}+\frac{1-\eta }{h_{1}^{3}+h_{2}^{3}}}}$$

while the corresponding stress in glass cover (*i* = 1) depends on the stress-effective thickness:

(6) $${\hat{h}}_{1;\sigma }=\sqrt{\frac{1}{\frac{2\eta h_{s;2}}{h_{1}^{3}+h_{2}^{3}+12\frac{h_{1}h_{2}}{h_{1}+h_{2}}H}+\frac{h_{1}}{{\hat{h}}_{w}^{3}}}}$$

with

(7) $$h_{s;2}=\frac{h_{2}H}{h_{1}+h_{2}}\;H=h_{int}+\frac{h_{1}+h_{2}}{2}$$

Similar to Equations (6) and (7), it is possible to express the stress-effective thickness for the backsheet (*i* = 2).

According to Figure 3, for the load-bearing capacity exploitation of the PV system, a key role is assigned to the actual shear bonding efficiency and out-of-plane bending rigidity of the sandwich system, where:

(8) $$0\leq \eta =\frac{1}{1+\frac{D_{1}+D_{2}}{\left(G_{int}/h_{int}\right)D_{tot}}\frac{12D_{1}D_{2}}{D_{1}h_{2}^{2}+D_{2}h_{1}^{2}}\Psi }\leq 1$$

with

(9) $$D_{tot}=D_{1}+D_{2}+\frac{12D_{1}D_{2}}{D_{1}h_{2}^{2}+D_{2}h_{2}^{2}}$$

(10) $$D_{i}=\frac{E_{i}h_{i}^{3}}{12\left(1-\nu_{i}^{2}\right)}\;(i=1,2)$$

and

(11) $$D_{abs}=D_{1}+D_{2}$$

The nondimensional coefficient *η* depends on the shear stiffness of EVA interlayer (*G_int_*) and its thickness (*h_int_*), but also on the plate geometry, its cross-section layout, and on the boundary and loading conditions (based on Ψ [31,32]).

## 3. Analysis of Analytical Results

### 3.1. Encapsulant Stiffness and Shear Coupling

**Figure 5:**
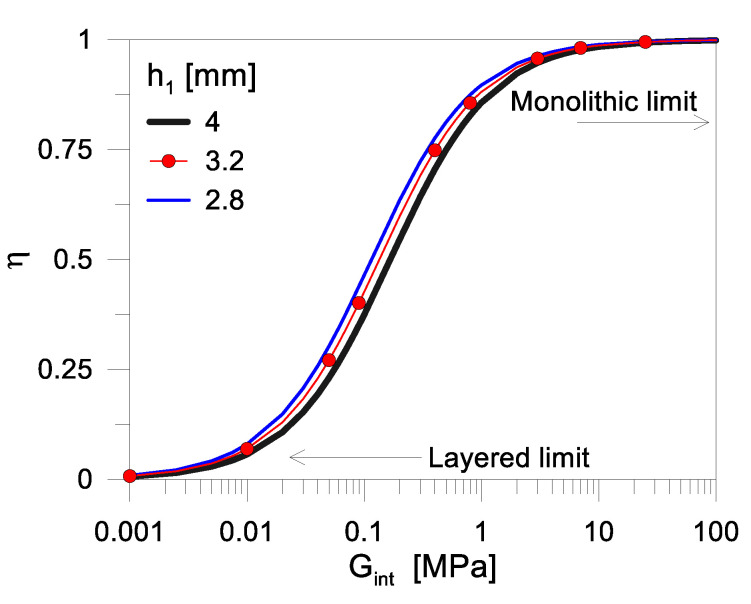
Calculated shear bond efficiency, in terms of variation of the shear coupling parameter *η* (0 ≤ *η* ≤ 1 based on Equation (8)) as a function of *G_int_*, for a simply supported PV module.

Figure 5 shows the typical trend of the shear coupling parameter *η* given in Equation (8), as a function of *G_int_*, by varying the cross-sectional layout of the resisting section. In particular, *h*_1_ = 3.2 mm is set as the reference glass thickness in present study (Table 1), and further minor modifications are considered in the cover thickness (*h*_1_ = 2.8 mm and 4 mm, respectively), while the other section features are kept fixed as indicated in Section 2.2.

As shown in Figure 5, there is a marked and relatively fast transition of *η* in the conventional range comprised between the “layered” bond (*η* = 0) and the ideal “monolithic” condition of fully rigid connection (*η* = 1), which is of major interest from both a theoretical and practical point of view. The modification of shear rigidity *G_int_* between ≈0.01 MPa and ≈10 MPa reveals a major effect in terms of *η*.

The mechanical problem may be not originated by the type of interlayer in use to encapsulate the solar cells, but by its viscoelastic behaviour and by the corresponding *G_int_* modification under variable external conditions. Under this assumption, even a minor variation in the cross-sectional layout of the PV module (in particular the glass or backsheet thickness) can have relevant mechanical effects for out-of-plane bending considerations. As far as a possible reduction in the shear bond adhesion of the encapsulant also takes place (i.e., in addition/in combination with the viscoelastic modification of material stiffness), the *η* parameter as in Figure 5 can further rapidly decrease and thus manifest in a global, progressively degrading mechanical response of the PV sandwich system.

### 3.2. Encapsulant Stiffness and Mechanical Capacity

While glass rigidity is not affected by long-term phenomena [42], the encapsulant stiffness has major direct effects in terms of distribution of internal actions (i.e., Figure 2 and Figure 3), and thus in terms of stress peaks in the glass and backsheet layers that should be verified based on Equations (3) and (4). As far as the shear bonding is weak ($G_{int}\to 0$), the “layered” behaviour of Figure 3 takes place and most of the external load is sustained by the glass cover only. Contrarily, an efficient shear bond results in a more effective composite action for the PV section, and thus in a more pronounced load-bearing contribution of the backsheet in bending, with a stress and deflection peak reduction in glass.

**Figure 6:**
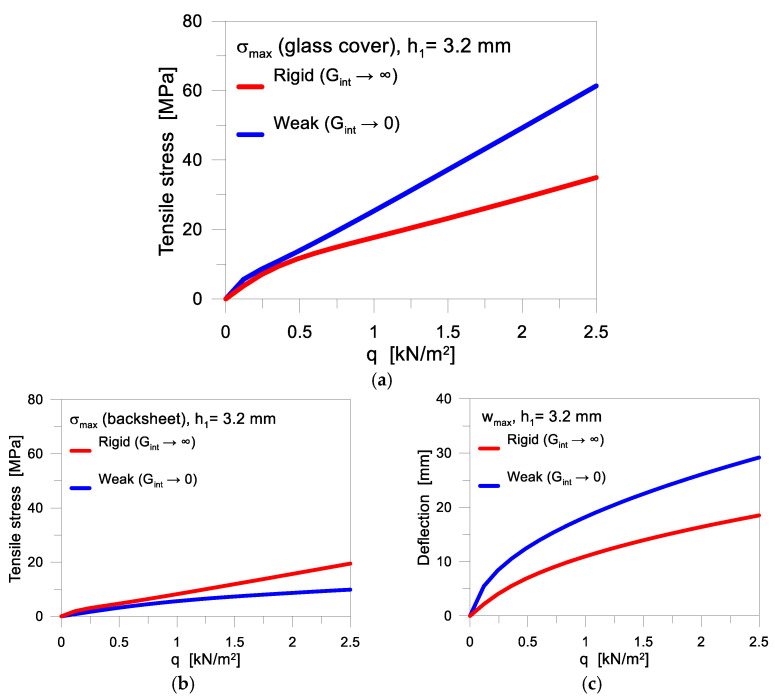
Mechanical analysis of the quasi-static performance of PV module, as a function of the encapsulant stiffness *G_int_*: stress peaks *σ_max_* in (**a**) glass cover and (**b**) backsheet, with (**c**) corresponding deflection *w_max_* for the monolithic (*G_int_* → ∞) or layered configurations (*G_int_* → 0).

Typical analytical trends are shown in Figure 6 in terms of (a) glass or (b) backsheet stress peaks and (c) deflection, respectively. As shown, a rigid shear bond allows a more balanced distribution of internal stresses in both the glass cover and in the backsheet, tending to the ideal “monolithic” setup of Figure 3.

The corresponding deflection (Figure 6c) is further mitigated by the efficient composite action, and can possibly satisfy the limit deformation for glass as in Equation (4). When the PV module suffers for a relatively weak shear bond, in contrast, most of the beneficial composite action fully vanishes, and the applied mechanical loads are mainly sustained by the glass cover only (Figure 6a). A minimum contribution in the overall load-bearing capacity derives from the backsheet, and the “layered” bending performance results in a marked increase of the corresponding deflection (Figure 6c).

### 3.3. Minimum Shear Bond Efficiency Detection

Assuming that the load-bearing capacity assessment of a PV module like in Figure 4 is governed by the most severe of limiting conditions like Equations (3) and (4), the optimal mechanical performance of the PV system can be further exploited and mitigated.

**Figure 7:**
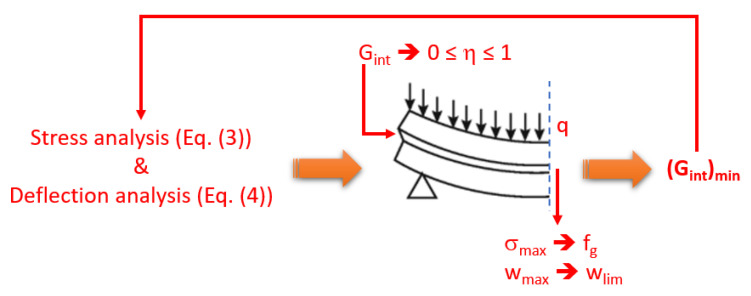
Minimum shear bond efficiency estimation for the mechanical performance verification of PV modules under quasi-static mechanical loads.

According to Figure 7, the typically high out-of-plane bending flexibility that derives from properties indicated in Table 1 manifests in stress peaks *σ_max_* and measured deflections *w_max_* under external pressures *q*, which could be further magnified by any deterioration in the shear coupling of the encapsulant (i.e., Figure 5).

It is thus important to verify the minimum shear coupling efficiency to preserve the PV module integrity and functionality.

**Figure 8:**
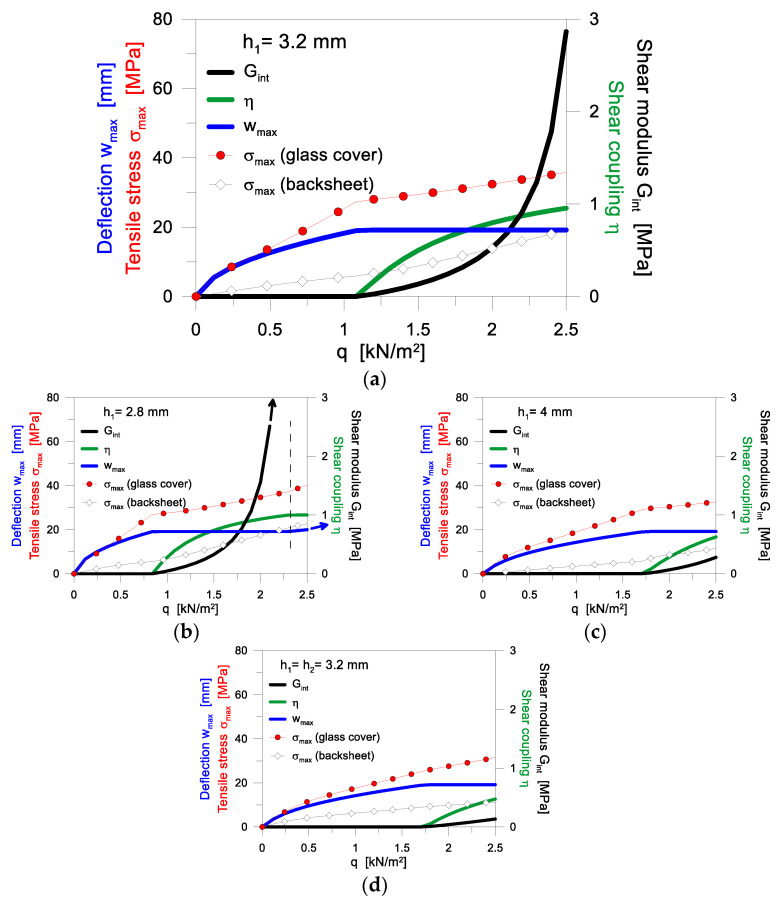
Mechanical analysis of the quasi-static response of PV module, as a function of the encapsulant stiffness. Stress peaks in glass cover, backsheet, deflection and shear bond efficiency varying the thickness of glass: (**a**) *h*_1_ = 3.2 mm; (**b**) *h*_1_ = 2.8 mm; (**c**) *h*_1_ = 4 mm (with *h*_2_ = 1 mm and *h_int_* = 1 mm); (**d**) *h*_1_ = *h*_2_ = 3.2 mm (with *h_int_* = 1 mm).

Figure 8, in this regard, shows the minimum bond efficiency (i.e., minimum shear modulus *G_int_* or minimum shear coupling *η*) that is required to satisfy the deflection limit for glass as in Equation (4), under an assigned uniform pressure *q*. In the present analysis, it is assumed that *w_max_* =*w_lim_* = *b*/60 is the limit deflection. The corresponding tensile stress peaks (both in the glass cover and in the backsheet) are also shown in Figure 8, as a function of the imposed uniform pressure *q*.

It can be noted in Figure 8a that—to satisfy the limit deflection requirement of Equation (4)—the reference cross-section composition/layout requires a very rigid shear bond (i.e., at least *η* ≈ 0.8) for an imposed pressure *q* > 1.2 kN/m^2^. This confirms that the mechanical test certification as in Figure 1 is highly demanding for PV composite systems and secondary components. In the present analysis, such a strong shear bond efficiency corresponds to a still relatively low shear modulus of the encapsulant (*G_int_* ≈ 0.5 MPa in Figure 8a, when *q* ≈ 1.2 kN/m^2^). However, the minimum stiffness value is also further affected by many other aspects, such as the cross-sectional layout (Figure 8b–d).

Moreover, Figure 8 shows the maximum stress peaks, derived from Equation (3), which should be verified for the glass cover or the backsheet. Notably, as seen in Figure 8b, a thinner glass cover (*h*_1_ = 2.8 mm) strongly affects the bending capacity of the PV system, especially for *q* > 2 kN/m^2^, while a minimum thickness increase (*h*_1_= 4 mm in Figure 8c) can efficiently contribute to the composite action in bending, even with a weak shear bonding. For higher loads, however, it is worth observing that even a rigid shear bond would not be able to satisfy the imposed deflection limitation, as can be seen in Figure 8b for *q* > 2.3 kN/m^2^. Finally, see Figure 8d, a symmetric cross-section layout (*h*_1_ = *h*_2_ = 3.2 mm) would largely increase the out-of-plane bending stiffness and load-bearing capacity of the PV module, even with a relatively weak shear bond.

### 3.4. Interfacial Stress Peaks

As far as the out-of-plane bending stiffness (and thus the minimum stiffness requirement to satisfy the limit on *w_max_*) modifies the examined PV system, the expected stress (and strain) at the encapsulant interfaces (and thus in the embedded solar cells) also progressively modifies and requires specific verifications. The stress demand in the bonding surfaces can be analytically quantified on the base of the corresponding stress-effective thickness [31], which is defined according to Section 2.5:

(12) $${\hat{h}}_{int,1;\sigma }=\sqrt{\frac{1}{\left|\frac{2\eta h_{s;2}}{h_{1}^{3}+h_{2}^{3}+12\frac{h_{1}h_{2}}{h_{1}+h_{2}}H}-\frac{h_{1}}{{\hat{h}}_{w}^{3}}\right|}}$$

(13) $${\hat{h}}_{int,2;\sigma }=\sqrt{\frac{1}{\left|\frac{2\eta h_{s;1}}{h_{1}^{3}+h_{2}^{3}+12\frac{h_{1}h_{2}}{h_{1}+h_{2}}H}-\frac{h_{2}}{{\hat{h}}_{w}^{3}}\right|}}$$

and analysed to assess possible delamination phenomena in terms of mechanical quasi-static response of the PV system [43,44]. Iin Equations (13) and (14), the subscripts “1” or “2” denote the interface of encapsulant film with “layer 1” (glass cover) or “layer 2” (backsheet), respectively.

### 3.5. Dynamic Bending Stiffness

The shear bond efficiency of the encapsulant in use has implicit major effects on the quasi-static mechanical performance of a given PV system, and in a similar way also affects its dynamic response under design loads. In this regard, it is worth the reminder that flexible systems are particularly susceptible to possible dynamic and aeroelastic interaction with wind loads.

As a matter of fact, the effective out-of-plane bending stiffness of the PV sandwich section in Figure 4 depends on the shear coupling parameter *η* of Figure 5. This means that—under well-defined time-loading and temperature conditions—the *G_int_* value and the corresponding *η* parameter also have consequences in terms of dynamic features.

**Figure 9:**
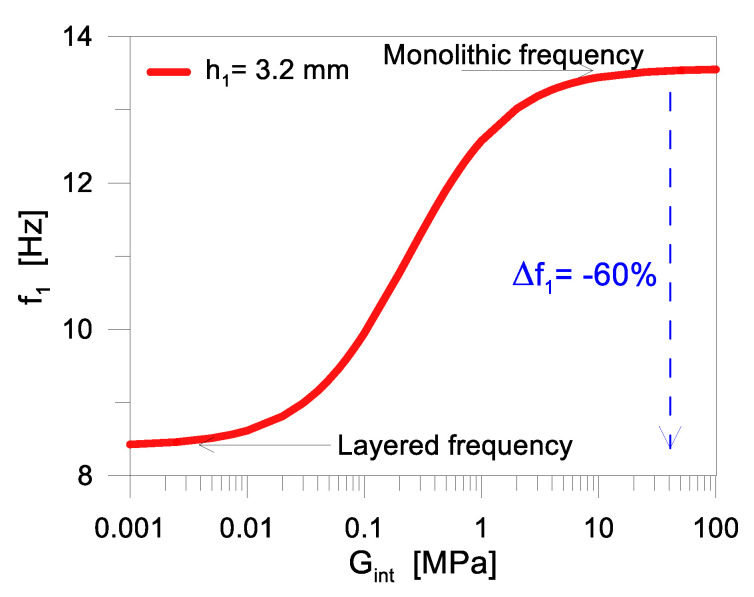
Calculated fundamental vibration frequency *f*_1_ for the examined PV module, as a function of the encapsulant stiffness *G_int_* (based on Equation (14)).

As an example, Figure 9 shows the modification of the fundamental vibration frequency *f*_1_ for the examined PV module (with four edges simply supported) as a function of *G_int_*, where [46]:

(14) $$f_{1}=\frac{\omega_{1}}{2\pi }=\frac{\pi }{2}\left(\frac{1}{L^{2}}+\frac{1}{B^{2}}\right)\sqrt{\frac{D_{eff}}{\bar{m}}}$$

In Equation (14), $D_{eff}$ is the actual out-of-plane bending stiffness of the PV system and $\bar{m}$ is its weight per unit of area. Reasonably, it is accepted that:

- $D_{eff}\to D_{abs}$ when $G_{int}\to 0$ and thus η→0 (layered limit), and
- $D_{eff}\to D_{tot}$ with a rigid shear coupling ($G_{int}\to \infty$ and η→1 at the monolithic limit).

Accordingly, *f*_1_= *f*(*G_int_*) in Equation (14) is comprised between two limit bounds (Figure 9).

The transition between the “monolithic” and “layered” bounds, as shown in Figure 9, is associated with a vibration frequency decrease down to −60%, for the examined configuration. Assured that the present analytical results have major effects on the PV module efficiency (i.e., the integrity of the electrical components that are part of the PV sandwich section), it is clear that its dynamic mechanical performance should be in general addressed with a special attention for the shear coupling efficiency of its load-bearing components. More in detail, a given wind pressure would induce different stress/deflection demands in the glass cover and backsheet, as well as in the electrical components.

With the reminder that the shear bond efficiency is implicitly associated with the “mechanical” and “layered” limits of Figure 3, the frequency trend in Figure 9 suggests some important outcomes. In particular:

- A dynamic/impulsive mechanical load typically corresponds to a stiffer response of the viscoelastic encapsulant. The shorter is the time-loading, and the stiffer is the shear bond, thus the PV module bending response ideally tends to the “monolithic” bound. Structurally speaking, the glass cover can benefit from a more pronounced composite action of the sandwich section, with more uniform distribution of internal stresses. It is expected that the maximum deflection is also reduced, thanks to the increased bending stiffness of the PV module.
- A long-term mechanical load is associated with the progressive relaxation of the viscoelastic encapsulant, which is implicitly associated with a bending stiffness loss for the PV model as a whole, with a mechanical behaviour similar to the “layered” configuration.
- All the intermediate scenarios are characterized by a bending performance that is associated with important modifications in the shear bond efficiency and composite action of the PV section, with major effects in the load-bearing and electrical components.

### 3.6. Mechanical Boundaries and Load Distribution

**Figure 10:**
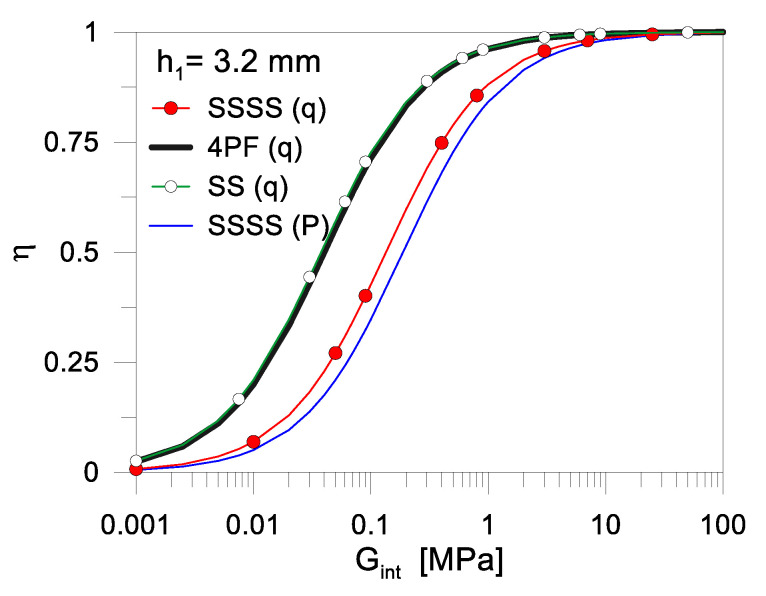
Calculated shear bond efficiency, in terms of variation of the shear coupling parameter *η* (0 ≤ *η* ≤ 1 based on Equation (8)) as a function of *G_int_*, for a simply supported PV module under various loading and boundary conditions.

A final remark goes to the boundary and loading configuration for the examined PV modules. As far as their load-bearing mechanical performance depends on Equations (3) and (4), the mechanical boundaries and loading conditions have implicit effects on the composite action of the constituent layers. Major sensitivities of these performance indicators are also expected in the long-term period. Figure 10 shows the typical *η* variation for the examined case-study system, as a function of *G_int_*. The reference configuration is detected as “SSSS (q)”, which well agrees with Figure 2a. For practical comparisons, the same PV sandwich section is also investigated in terms of expected composite action by considering three further configurations, namely:

- “SS (q)”: two short edges supported (with long edges unrestrained) and a uniformly distributed load *q*;
- “4PF (q)”: four point-fixing restraints at the corners of the PV module, with uniformly distribute load *q*;
- “SSSS (P)”: four simply supported edges and a mechanical load on a central 10 × 10 cm surface.

While the metal frame and the electrical components of the PV module are still disregarded in the analysis of present comparative results (but can be implicitly verified through an appropriate deflection limit), a marked sensitivity of shear bonding efficiency can be seen, and thus a further modification in the expected composite action of the sandwich system, which reflects in a different out-of-plane bending performance and global deflection. The latter could thus be efficiently fixed in a rational amplitude (i.e., *w_lim_* = *b*/60, or even lower, to prevent micro-cracks in solar cells) to implicitly preserve the electrical functionality of the system, and/or even guide or optimize a more refined numerical and experimental analysis of the composite section.

## 4. Conclusions

The mechanical characterization of photovoltaic (PV) and building integrated photovoltaic (BIPV) solutions is a critical step for their functionality optimization, and requires special attention and procedural steps. In this paper, primary attention was given to the analytical analysis of the mechanical role of the thin glass cover, and its contribution in the composite action of PV modules when affected by possible modification in the shear coupling and shear rigidity of the Ethylene-Vinyl Acetate (EVA) encapsulant.

The encapsulant is not only required to protect the embedded solar cells and the electrical components of a given PV module, but also to ensure a minimum composite action and out-of-plane bending capacity to the sandwich system, which is based on the structural interaction of glass and/or plastic layers.

All the above recalled aspects are mutually affected by each other, and should be maximized for the product optimization, both for short-term and long-term scenarios.

To this aim, a parametric analytical analysis was presented in this paper, based on consolidated approaches in use for the design and verification of laminated glass plates. The analysis, as shown, posed the attention a selection of configurations of technical interest, by exploring the shear coupling efficiency of the viscoelastic film. Its effects—in conjunction with further cross-sectional geometrical aspects—were investigated in terms of stress and deformation demands for commercial PV modules subjected to quasi-static and dynamic mechanical loads. In particular, the analysis allowed quantification of the minimum shear coupling that is required to satisfy the glass cover verification (and thus implicitly preserve the electrical components of PV modules). Also, the dynamic analysis of commercial PV modules highlighted that the encapsulant stiffness can affect the out-of-plane bending stiffness of the resisting cross-section up to around a 60% variation, within the well-known “monolithic” and “layered” configurations.

Whilst the current study explored the global mechanical performance of typical commercial single-glass PV modules, the presented results showed that the preliminary limitation of their maximum deflection can implicitly preserve the functionality of solar cells, and thus allow optimization of a more refined and expensive experimental or Finite Element numerical analysis of these composite systems.

## Figures and Tables

**Table 1:** Reference geometrical and mechanical properties for the examined PV module: thickness (*h*), modulus of elasticity (*E*), Poisson’ ratio (*ν*) and material density (*r*).

		Glass (Cover)	Tedlar^®^ (Backsheet)	EVA (Encapsulant)	Solar Cells (Disregarded)	Aluminium Frame (Disregarded)
*h*	[mm]	3.2	1	1	0.2	Var. (Figure 4a)
*E*	[GPa]	70	25	0.005 (var.)	170	69
*ν*	-	0.23	0.3	0.49	0.25	0.3
*r*	[kg/m^3^]	2490	1000	950	2330	2700

## Data Availability

Data are contained within the article.

## References

[1] Sander M., Dietrich S., Pander M., Ebert M., Bagdahn J. (2013). Systematic investigation of cracks in encapsulated solar cells after mechanical loading. Sol. Energy Mater. Sol. Cells.

[2] Dietrich S., Pander M., Sander M., Ebert M. (2013). Mechanical Investigations on Metallization Layouts of Solar Cells with Respect to Module Reliability. Energy Procedia.

[3] Dabaghzadeh N., Eslami M. (2019). Temperature distribution in a photovoltaic module at various mounting and wind conditions: A complete CFD modelling. J. Renew. Sustain. Energy.

[4] Li Y., Xie L., Zhang T., Wu Y., Sun Y., Ni Z., Zhang J., He B., Zhao P. (2020). Mechanical analysis of photovoltaic panels with various boundary condition. Renew. Energy.

[5] Hartley J.Y., Owen-Bellini M., Truman T., Maes A., Elce E., Ward A., Khraishi T., Roberts S.A. (2020). Effects of Photovoltaic Module Materials and Design on Module Deformation Under Load. IEEE J. Photovolt..

[6] Corrado M., Infuso A., Paggi M. (2017). Simulated hail impacts on flexible photovoltaic laminates: Testing and modelling. Meccanica.

[7] Chakraborty S., Haldkar A.K., Kumar N.M. (2023). Analysis of the hail impacts on the performance of commercially available photovoltaic modules of varying front glass thickness. Renew. Energy.

[8] Gong J., Xie L., Li Y., Ni Z., Wei Q., Wu Y., Cheng H. (2021). Analysis of the Impact Resistance of Photovoltaic Panels Based on the Effective Thickness Method. J. Renew. Mater..

[9] (2021). Terrestrial Photovoltaic (PV) Modules—Design Qualification and Type Approval—Part 2: Test Procedures.

[10] Shah N.A., Gul R.M., Khan Z.H. (2022). Mechanical Integrity and Failure Analysis of Photovoltaic Modules under Simulated Snow Loads Using Pneumatic Airbag Setup. J. Power Energy Eng..

[11] Papargyri L., Papanastasiou P., Georghiou G.E. (2022). Effect of materials and design on PV cracking under mechanical loading. Renew. Energy.

[12] Dietrich S., Pander M., Sander M., Schulze S.-H., Ebert M. Mechanical and Thermo-Mechanical Assessment of Encapsulated Solar Cells by Finite-Element-Simulation. Proceedings of the SPIE—The International Society for Optical Engineering—Reliability of Photovoltaic Cells, Modules, Components, and Systems III.

[13] Haghi M., Aßmus M., Naumenko K., Altenbach H. (2018). Mechanical Models and Finite-Element Approaches for the Structural Analysis of Photovoltaic Composite Structures: A Comparative Study. Mech. Compos. Mater..

[14] Aßmus M., Bergmann S., Naumenko K., Altenbach H. (2017). Mechanical behaviour of photovoltaic composite structures: A parameter study on the influence of geometric dimensions and material properties under static loading. Compos. Commun..

[15] Naumenko K., Eremeyev V.A. (2014). A layer-wise theory for laminated glass and photovoltaic panels. Compos. Struct..

[16] Li G., Wang F., Feng F., Wei B. (2022). Hot Spot Detection of Photovoltaic Module Based on Distributed Fiber Bragg Grating Sensor. Sensors.

[17] Dadaniya A., Datla N.V. (2020). Degradation prediction of encapsulant-glass adhesion in the photovoltaic module under outdoor and accelerated exposures. Sol. Energy.

[18] Dobra T., Vollprecht D., Pomberger R. (2022). Thermal delamination of end-of-life crystalline silicon photovoltaic modules. Waste Manag. Res..

[19] Sangpongsanont Y., Chuangchote S., Chenvidhya D., Kirtikara K. (2023). Annual expansion in delamination of front encapsulant in tropical climate Field-Operated PV modules. Sol. Energy.

[20] Meena R., Pareek A., Gupta R. (2024). A comprehensive Review on interfacial delamination in photovoltaic modules. Renew. Sustain. Energy Rev..

[21] Jaszczur M., Hassan Q., Teneta J., Majewska E., Zych M. (2018). An analysis of temperature distribution in solar photovoltaic module under various environmental conditions. MATEC Web. Conf..

[22] Repins I.L., Jordan D.C., Woodhouse M., Theristis M., Stein J.S., Seigneur H.P., Colvin D.J., Karas J.F., McPherson A.N., Deline C. (2023). Long-term impact of light- and elevated temperature-induced degradation on photovoltaic arrays. MRS Bull..

[23] Theristis M., Stein J.S., Deline C., Jordan D., Robinson C., Sekulic W., Anderberg A., Colvin D.J., Walters J., Seigneur H. (2022). Onymous early-life performance degradation analysis of recent photovoltaic module technologies. Prog. Photovolt. Res. Appl..

[24] Romer P., Pethani K.B., Beinert A.J. (2024). Effect of inhomogeneous loads on the mechanics of PV modules. Prog. Photovolt. Res. Appl..

[25] Pander M., Dietrich S., Schulze S.-H., Eitner U., Ebert M. Thermo-mechanical assessment of solar cell displacement with respect to the viscoelastic behaviour of the encapsulant. Proceedings of the 12th International Conference on Thermal, Mechanical & Multi-Physics Simulation and Experiments in Microelectronics and Microsystems.

[26] Rodrigues T., Jordão S., Bedon C. Long-term effects on structural glass beam. Proceedings of the XI Congresso de Construção Metálica e Mista.

[27] Nielsen J.H., Jónsson B.G., Bedon C. (2020). A novel concept for a reinforced glass beam carrying long term loads. Glas. Struct. Eng..

[28] Makrides G., Theristis M., Bratcher J., Pratt J., Georghiou G.E. (2018). Five-year performance and reliability analysis of monocrystalline photovoltaic modules with different backsheet materials. Sol. Energy,.

[29] Bedon C. (2019). Diagnostic analysis and dynamic identification of a glass suspension footbridge via on-site vibration experiments and FE numerical modelling. Compos.Struct..

[30] Bedon C. (2019). Issues on the Vibration Analysis of In-Service Laminated Glass Structures: Analytical, Experimental and Numerical Investigations on Delaminated Beams. Appl. Sci..

[31] (2013). Istruzioni Per la Progettazione, L’esecuzione ed il Controllo di Costruzioni con Elementi Strutturali di Vetro.

[32] Galuppi L., Royer-Carfagni G. (2012). The effective thickness of laminated glass plates. J. Mech. Mater. Struct..

[33] Serafinavičius T., Lebet J.-P., Louter C., Lenkimas T., Kuranovas A. (2013). Long-term Laminated Glass Four Point Bending Test with PVB, EVA and SG Interlayers at Different Temperatures. Procedia Eng..

[34] Paggi M., Sapora A. (2015). An Accurate Thermoviscoelastic Rheological Model for Ethylene Vinyl Acetate Based on Fractional Calculus. Int. J. Photoenergy.

[35] Andreozzi L., Bati S.B., Fagone M., Ranocchiai G., Zulli F. (2015). Weathering action on thermo-viscoelastic properties of polymer interlayers for laminated glass. Constr. Build. Mater..

[36] Antolinc D., Belis J.L.I.S., Bos F.P., Louter P.C. (2020). Three-Point Bending Test of Laminated Glass With PVB and EVA Interlayers at Elevated Temperature. Proceedings of the Challenging Glass 7—Conference on Architectural and Structural Applications of Glass.

[37] Bornemann S., Henning S., Naumenko K., Pander M., Thavayogarajah N., Würkner M. (2022). Strength analysis of laminated glass /EVA interfaces: Microstructure, peel force and energy of adhesion. Compos. Struct..

[38] Knight J.T., El-Sisi A.A., Elbelbisi A.H., Newberry M., Salim H.A. (2022). Mechanical Behavior of Laminated Glass Polymer Interlayer Subjected to Environmental Effects. Polymers.

[39] (2004). Glass in buildings—Basic Soda Lime Silicate Glass Products.

[40] (2016). Aluminium and Aluminium Alloys—Sheet, Strip and Plate—Part 2: Mechanical Properties.

[41] Tedlar Technical Bulletin (2020). Mechanical Properties of Tedlar Films.

[42] Udi U.J., Yussof M.M., Ayagi K.M., Bedon C., Kamarudin M.K. (2023). Environmental degradation of structural glass systems: A review of experimental research and main influencing parameters. Ain Shams Eng. J..

[43] Zhang T., Xie L., Li Y., Mallick T.K., Wei Q., Hao X., He B. (2018). Experimental and Theoretical Research on Bending Behavior of Photovoltaic Panels with a Special Boundary Condition. Energies.

[44] Tummalieh A., Beinert A.J., Reichel C., Mittag M., Neuhaus H. (2022). Holistic design improvement of the PV module frame: Mechanical, optoelectrical, cost, and life cycle analysis. Prog. Photovolt. Res. Appl..

[45] Kontges M., Siebert M., Morlier A., Illing R., Bessing N., Wegert F. (2016). Impact of transportation on silicon wafer-based PV modules. Prog. Photovolt. Res. Appl..

[46] Clough R.W., Penzien J. (1993). Dynamics of Structures.

